# Iron administered in the neonatal period changed memory, brain monoamine levels, and BDNF mRNA expression in adult Sprague–Dawley rats

**DOI:** 10.1007/s43440-024-00626-0

**Published:** 2024-07-16

**Authors:** Zofia Rogóż, Kinga Kamińska, Elżbieta Lorenc-Koci, Agnieszka Wąsik

**Affiliations:** 1 Department of Pharmacology, Maj Institute of Pharmacology Polish Academy of Sciences, Kraków, Poland; 2 Department of Neuropsychopharmacology, Maj Institute of Pharmacology Polish Academy of Sciences, Kraków, Poland; 3 Department of Neurochemistry, Maj Institute of Pharmacology Polish Academy of Sciences, Kraków, Poland

**Keywords:** Iron treatment in the neonatal period, Behavioral tests, Monoamine levels, BDNF mRNA expression, Rats

## Abstract

**Background:**

Iron is one of the key microelements in the mammalian body and is the most abundant metal in the brain. Iron, a very important chemical element in the body of mammals, is the most abundant metal in the brain. It participates in many chemical reactions taking place in the central nervous system acting as a cofactor in key enzymatic reactions involved in neurotransmitter synthesis and degradation, dendritic arborization, and myelination. Moreover, iron accumulation in the brain has been implicated in the pathogenesis of neurogenerative disorders.

**Material and methods:**

The aim of our study was to assess the influence of iron administered orally (30 mg/kg) to rats in the neonatal period (p12-p14) by testing the performance of rats in the open field and social interaction tests, and by evaluating the recognition memory, monoamine levels in some brain structures, and BDNF mRNA expression. The behavioral and biochemical tests were performed in adult p88-p92 rats.

**Results:**

Iron administered to rats in the neonatal period induced long-term deficits in behavioral tests in adult rats. It reduced the exploratory activity in the open field test. In the social interaction test, it induced deficits in the parameters studied, and decreased memory retention. Moreover, iron changed the brain monoamine levels in some studied brain structures and decreased the expression of BDNF mRNA in the hippocampus.

**Conclusions:**

All earlier and our present results indicated that iron administered to rats in the neonatal period induced an increase in oxidative stress which resulted in a change in the brain monoamine levels and decreased BDNF mRNA expression which may play a role in iron-induced memory impairment in adult rats.

## Introduction

It has been demonstrated that iron is one of the key microelements in the mammalian body and is the most abundant metal in the brain. Iron participates in many chemical reactions taking place in the central nervous system acting as a cofactor in key enzymatic reactions involved in neurotransmitter synthesis and degradation, dendritic arborization, and myelination [1, 2]. In addition, iron is a component of metalloproteins involved in oxygen transport and energy metabolism. On the other hand, ferrous iron can react with hydrogen peroxide in the Fenton reaction, producing a very reactive hydroxyl radical, which is a very dangerous compound causing damage to membrane proteins, lipids, and deoxyribonucleic acid (DNA) [3, 4].

The effects of iron deficiency have been well documented, while relatively little is known about the long-term consequences of iron overload during development. Some papers have indicated that concentrations of iron in the brain gradually increase during the aging process [5, 6], and undergoes selective accumulation in the brains of patients suffering from neurodegenerative diseases [7]. High levels of redox-active iron in the brain have been linked to the pathogenesis of neurodegenerative diseases such as Alzheimer's disease, Parkinson's disease, Huntington’s disease and other diseases [8], however, the gradual increase in iron levels in the brain appears to be a feature of normal aging. Excessive brain iron concentrations may result from the consumption of infant formula supplemented with additional iron, thereby altering iron absorption pathways in the brain and increasing the risk of iron-related neurodegeneration in later life.

Interestingly, recent studies have indicated that iron amount in brain structures positively correlated with poorer cognitive test scores in Alzheimer’s patients [9]. Furthermore, active iron concentration in the cerebrospinal fluid elevated with the degree of cognitive deficits from normal and mild cognitive impairment [10]. Moreover, it was demonstrated that pantothenate kinase-associated neurodegeneration (PKAN) is o type of neurogeneration with brain iron accumulation (NBIA). NBIA-related neurogeneration encompasses a clinically and genetically heterogeneous group of disorders observed in both children and adults. The NBIA phenotype typically begins in childhood or early adulthood and manifests with behavioral disturbances, motor impairments, and dementia. In the early stages of the disease, in addition to spasticity and parkinsonism, neuropsychiatric problems such as emotional instability, compulsions and impulsivity, depression or anxiety are often observed. However, later in the disease, hallucinations may occur. In the case of diseases developing the phenotype in childhood, in addition to cognitive impairment and motor axonal neuropathy may also occur. In the case of late-onset diseases, i.e. those with onset in adults, neuropsychiatric disorders predominate, including psychosis and dementia. This later-onset mitochondrial membrane protein-associated form (MPAN) can progress at an aggressive and accelerated rate, usually leading to death within 5–10 years [11–14]. Two types of PKAN phenotypic spectrum has been distinguished, namely classic PKAN and atypical PKAN. Classic PKAN appears in early childhood and is characterized by progressive dystonia, dysarthria, rigidity, and choreoathetosis. In contrast, atypical PKAN is characterized by later onset (age > 10 years), significant speech defects, psychiatric disorders and gradual progression of the disease [11, 12].

In addition, preclinical studies have shown that iron administered to rodents during early postnatal brain development induces memory deficits (e.g., spatial memory assessed using the eight-arm radial maze and aversive memory assessed using an inhibitory avoidance task) that are relevant to neurodegenerative diseases [15]. Moreover, neonatal iron treatment causes increased oxidative stress which leads to impaired recognition memory [16–18].

It has been demonstrated that male Wistar pups received vehicle or 10 mg/kg of Fe^2+^ orally a postnatal days 5–7, 12–14, 19–21 or 30–32. And then the long-term effects of iron on memory and parameters of oxidative stress in brain regions (hippocampus, cortex, substantia nigra and striatum) related to memory were tested. Only a group received iron from postnatal days 12–14 demonstrated memory blockade. Moreover, perinatal iron treatment caused oxidative damage in the brain and elevation superoxide production in mitochondria. Which evoked cognitive impairment in adult rats. These finding confirm the data that oxidative stress may lead important to the cognitive disturbances observed in normal ageing [16]. In light of the above data, our study aimed to evaluate the influence of iron administered to rats in the early postnatal brain development on performance in behavioral tests, e.g. memory deficits in the novel object recognition test, social interaction test, and open field test and on the monoamine levels in the main brain structures involved in the regulation of working memory: the frontal cortex and hippocampus as well as in the striatum [19–22], and on the brain-derived neurotrophic factor (BDNF) mRNA expression in 92-day-old rats.

## Materials and methods

### Animals and treatment

Pregnant Sprague–Dawley females at embryonic day 16 delivered by Charles River Company (Sulzfeld, Germany) were kept in individual cages under standard laboratory conditions; at room temperature of 21 ± 1 °C with 40–50% humidity, 12/12 h light/dark cycle (lights on from 7 am, lights off from 7 pm), with free access to standard laboratory chow and tap water. One day after birth, the sex of the pups was determined, and only males were left with their mothers to be used in further experimental procedures. Between the postnatal days p12 and p14 male Sprague–Dawley pups were administered orally either a single daily dose of 30 mg/kg of Fe^2+^ (iron carbonyl) (n = 12) or vehicle (5% sorbitol in water) in the control group (n = 12). Behavioral tests (open field, social interaction, and novel object recognition) evaluating the expression of changes in behavioral experiments were carried out in adulthood (at p88-91 days of age). The tissue (hippocampus, frontal cortex, and striatum) for biochemical assays was dissected on p92.

### Drugs and treatment

Iron (carbonyl iron, Fe^2+^, Sigma Aldrich, Saint Louis, MO, USA, Chemie GmbH, Kappelweg 1, D-91625 Schnelldorf, Germany, no C3518, 30 mg/kg, *po*). was dissolved in 5% sorbitol in water. The dose of the drug used in the present study was selected based on earlier publications [15, 23].

### Open field test

Exploratory activity was assessed in the open field test. For details see [24]. The open field test was performed on day p88. Each group consisted of 12 rats.

### Social interaction test

The social interaction test was performed as described in [24]. The social interaction test was performed on day p90 [24]. Each group consisted of twelve animals (six pairs).

### Novel object recognition test

The novel object recognition test was as described in [24]. The novel object recognition test was performed on day p91 [24]. Each group was composed of 12 rats.

### Biochemical analysis of monoamine and their metabolite concentrations

The tissue (frontal cortex [Fcx], hippocampus [HIP], and striatum [STR]) for biochemical assays was dissected on p92. Fcx, HIP, and STR were dissected and frozen on solid CO2 (− 70 °C) and stored until biochemical assays. Dopamine (DA) and its metabolites, 3,4-dihydroxyphenylacetic acid (DOPAC), 3-methoxytyramine (3-MT) and the final metabolite, homovanillic acid (HVA); serotonin (5-HT) and its metabolite 5-hydroxyindoleacetic acid (5-HIAA); noradrenaline (NA) and its metabolite normetanephrine (NM) were assayed using high-performance liquid chromatography (HPLC) with electrochemical detection. The chromatograph (HP 1050; Hewlett-Packard, Golden, CO, USA) was equipped with C18 columns. The procedure of sample preparation is based on our previous protocol [21]. Each group consisted of 10–12 rats.

### BDNF mRNA expression analysis (real-time PCR)

The tissue (HIP and Fcx) for biochemical assays was dissected on p92. Freshly isolated rat tissues were stored at − 80 °C before the analysis. Total RNA was isolated using a commercially available Bead-Beat Total RNA Mini Kit (A&A Biotechnology, no 031-100BB, PL) according to the manufacturer’s instructions. After dissolving in water, RNA (1 μg) was reverse-transcribed to cDNA using High Capacity cDNA Reverse Transcription kit with RNase inhibitor and random hexamers (MultiScribe™, Applied Biosystems, Life Technologies, Carlsbad, CA, no 4368813, USA). The BDNF mRNA level was determined by Real-Time PCR using predesigned TaqMan Gene Expression Assays (Applied Biosystems, no 4331182, UK). Assay IDs for the genes examined were as follows: BDNF (Rn01484925_m1) and for reference gene HPRT1 (Rn01527840_m1). Amplification was carried out in a total volume of 10 μl (FCx). The mixture containing: 1 × FastStart Universal Probe Master (Rox) mix (Roche, no 45–4913949001 Germany), 900 nM TaqMan forward and reverse primers, and 250 nM of hydrolysis probe labeled with the fluorescent reporter dye FAM at the 5′-end and a quenching dye at the 3′-end and RNAse free water. We used 50 ng of cDNA for the PCR template, Real-time PCR was conducted using thermal cycler Quant Studio 3 (Thermo Fisher Scientific, Waltham, MA, USA) and thermal cycling conditions were: 2 min at 50 °C and 10 min at 95 °C followed by 40 cycles at 9 °C for 15 s and at 60 °C for 1 min [24]. Each group consisted of 10–12 rats.

### Statistical analysis

The behavioral and biochemical data were evaluated using a Student’s *t*-test except novel object recognition test (Fig. 3A, B) which were analysed by two-way ANOVA. The recognition index was calculated for each rat, and was expressed in percentages. The results are presented as the ± SEM (standard errors of the mean); they were considered statistically significant when *p* < 0.05.

## Results

### Open field test

**Fig. 1:**
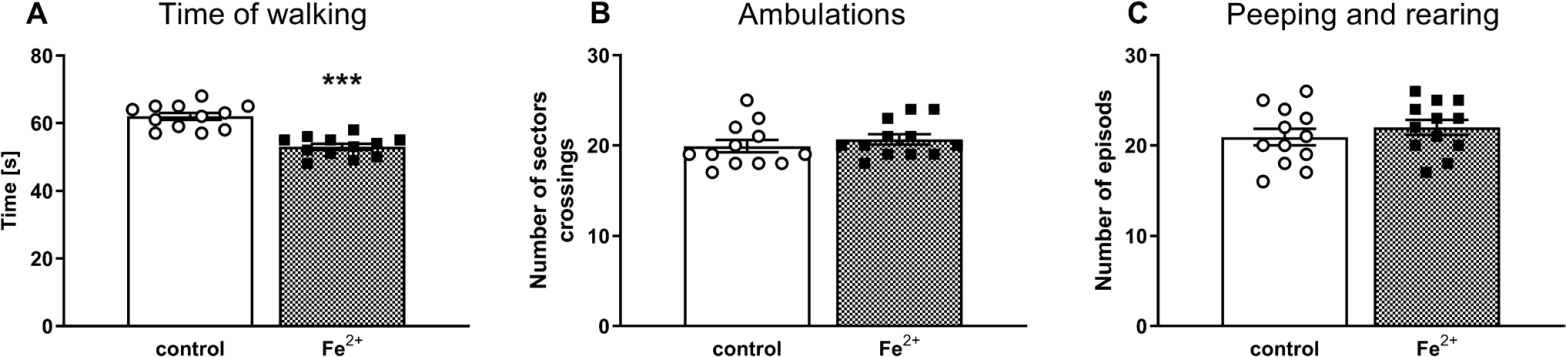
The effects of iron (Fe^2+^) administration on the exploratory activity in the open field test in the adult Sprague–Dawley rats. Between the postnatal days p12 and p14 male Sprague–Dawley pups were administered orally a single daily dose of 30 mg/kg of Fe^2+^ (iron carbonyl) or vehicle (control group). The exploratory activity in the open field test was examined in the adult 88 days old Sprague–Dawley rats; the time of walking, the number of sector crossing (ambulation), and the number of episodes of peeping under the edge of arena and rearing were assessed for 5 min. The results are expressed as the means ± SEM (n = 12 animals per group). Data were analyzed with a Student’s *t*-test. *** p < 0.001 *vs*. vehicle-treatment group

The exploratory activity in the open field test was evaluated as the time of walking, the number of sector crossings (ambulation), and the number of episodes of peeping and rearing on day p88. The Student’s *t*-test showed that in this test when pups were administered orally a single daily dose of iron (30 mg/kg) between the postnatal days p12 and p14, adult rats showed only a decrease in the time of walking by c.a. 15% (*t*_1,22_ = 43.46, *p* < 0.001), but the number of ambulation and peeping and rearing episodes was not changed (*t*_1,22_ = 0.69, ns) and (*t*_1,22_ = 0.77, ns), respectively (Fig. 1).

### Social interaction test

**Fig. 2:**
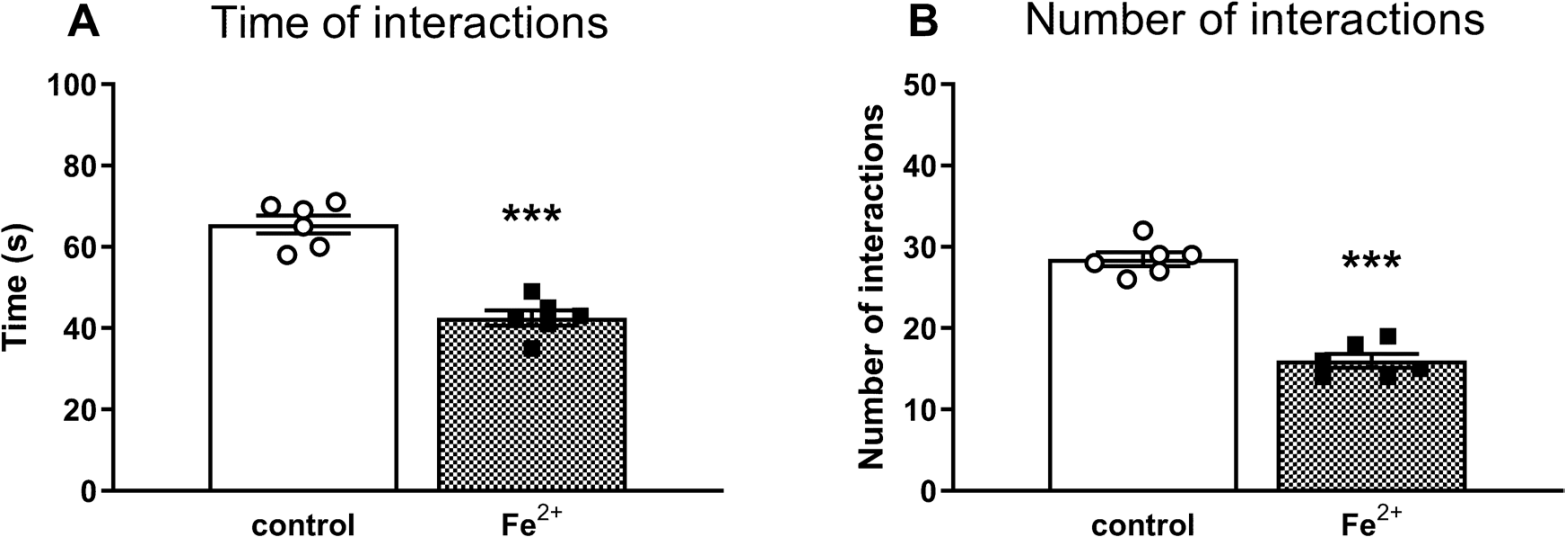
The effects of iron (Fe^2+^) administration in the social interaction test in the adult 90 days old Sprague–Dawley rats. Between the postnatal days p12 and p14 male Sprague–Dawley pups were administered orally a single daily dose of 30 mg/kg of Fe^2+^ (iron carbonyl) or vehicle (control group). Social interaction test performance in rats was assessed for 10 min by means of two parameters; **A** the total time spent in social interaction, **B** the number of these interactions. The results are shown as the mean ± SEM. Each group consisted of 6 pairs/group (12 rats). The statistically significant differences between the studied groups were calculated using a Student’s *t*-test. *** p < 0.001 *vs*. vehicle-treatment group

The Student’s *t*-test showed that in the social interaction test in rats, which between the postnatal days p12 and p14 were administered orally a single daily dose of iron (30 mg/kg), deficits in the studied parameters observed in adult rats included a decrease in the time of interactions and the number of episodes by 35 and 44% of the control rats (*t*_1,10_ = 61.75, *p* < 0.001) and (*t*_1,10_ = 107.76, *p* < 0.001), respectively (Fig. 2).

### Novel object recognition test

**Fig. 3:**
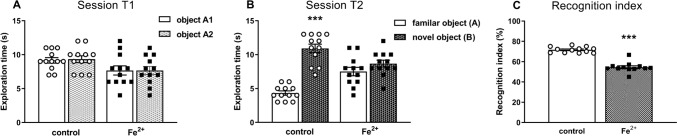
The effects of iron (Fe^2+^, iron carbonyl) administration in the novel object recognition test in the adult 91 days old Sprague–Dawley rats. The effect on the exploration time (s) in the introductory session T1 **(A)**. The effect on the exploration time (s) in the recognition session T2 **(B)**. Recognition index in the T2 session **(C)**. Recognition memory (T2 session) was tested 60 min after T1 (introductory session). The results are shown as the mean ± SEM. Each group consisted of 12 rats. The exploration of A1 and A2 objects in session T1 and the familiar and novel objects in session T2 was the subject of a separate analysis within each of the studied groups. The statistically significant differences between the studied groups were calculated using two-way ANOVA **A**, **B** and recognition index **C** was calculated by Student’s *t*-test. *** p < 0.001 *vs.* vehicle-treatment group

According to the two-way ANOVA, during T1, i.e. the introductory session, when pups received orally a single dose of vehicle or a single daily dose of iron (30 mg/kg) between the postnatal days p12 and p14, the adult animals spent a similar amount of time exploring the two identical objects (A1 and A2) (Fig. 3A). During T2, i.e. the recognition session (Fig. 3B), control rats spent significantly more time exploring the novel object (*F*_*1, 44*_ = *52,42*, *p* < 0.001), while rats receiving iron (30 mg/kg) showed no preference for a particular object (*t*_1,22_ = 0,77, ns). The Student’s *t*-test indicated that recognition index showed statistically significant differences between the exploration time of control rats and rats receiving iron (30 mg/kg), in the session T2 (*t*_1,22_ = 112.2, *p* < 0.001) (Fig. 3C).

### Biochemical analysis of monoamine and their metabolite concentrations

#### Dopamine (DA) and its metabolites in the Fcx

The Student’s *t*-test revealed an insignificant (*t*_1,20_ = 0.52, *ns*) effect of treatment on DA level in the Fcx of adult rats (Table 1). The same analysis showed an insignificant effect (*t*_1,21_ = 0.59, *ns*) on the DOPAC level, and also indicated an insignificant effect of treatment (*t*_1,20_ = 0.2, *ns*) on the 3-MT level (Table 1). A statistical analysis showed an insignificant (*t*_1,20_ = 0.77, *ns*) effect of treatment on HVA level in the Fcx (Table 1).

**Table 1:** The effects of iron (Fe^2+^) administration (30 mg/kg) on the dopamine (DA) metabolism in the frontal cortex of 92-day-old rats

Frontal cortex					
Treatment	n	DA	DOPAC	3-MT	HVA
Control	12	973 ± 201	372 ± 127	71 ± 21	119 ± 47
Fe^2+^	10	915 ± 206	401 ± 1 30	59 ± 20	114 ± 23
*t*-value		*t*_1,20_ = 0 .65 *ns*	*t*_1,21_ = 0.55 *ns*	*t*_1,20_ = 1.31 *ns*	*t*_1,20_ = 0.29 *ns*

The results are expressed as means ± SEM (n = 10–12 animals per group). Data were analyzed with a Student’s *t*-test. Between the postnatal days p12 and p14, male Sprague–Dawley pups were administered orally a single daily dose of vehicle (5% sorbitol of water, control group) or 30 mg/kg of body weight of Fe^2+^ (iron carbonyl). The 92-day-old rats were decapitated, and the tissue of the frontal cortex was dissected for biochemical assays

### Noradrenaline (NA) and its metabolite in the Fcx

The Student’s *t*-test showed a insignificant (*t*_1,21_ = 0.17, *ns*) effect of treatment on NA level in the Fcx of adult rats. At the same time, the effect of treatment on NM level was significant (*t*_1,20_ = 0.02, *p* < 0.05) (Table 2).

**Table 2:** The effects of iron (Fe^2+^) administration (30 mg/kg) on the noradrenaline (NA) and serotonin (5-HT) metabolism in the frontal cortex of 92- day-old rats

Frontal cortex					
Treatment	n	NA	NM	5-HT	5-HIAA
Control	12	174 ± 32	16 ± 3	123 ± 17	114 ± 7
Fe^2+^	10	201 ± 56	22 ± 8*	117 ± 34	122 ± 9
*t*-value		*t*_1,21_ = 1.42 *ns*	*t*_1,20_ = 2.59 *p* < 0.05	*t*_1,20_ = 0.48 *ns*	*t*_1,20_ = 0.77 *ns*

The results are expressed as means ± SEM (n = 10–12 animals per group). Data were analyzed with a Student’s *t*-test. Between the postnatal days p12 and p14, male Sprague–Dawley pups were administered orally a single daily dose of vehicle (5% sorbitol of water, control group) or 30 mg/kg of body weight of Fe^2+^ (iron carbonyl). The 92-day-old rats were decapitated, and the tissue of the frontal cortex was dissected for biochemical assays. Statistical significance: * p < 0.05 *vs.* control group

### Serotonin (5-HT) and its metabolite in the Fcx

The Student’s *t*-test revealed an insignificant (*t*_1,20_ = 0.48, *ns)* effect of treatment on 5-HT amount in the Fcx of adult rats. The same analysis showed an insignificant effect of treatment (*t*_1,20_ = 0.77, *ns*) on the 5-HIAA level*.* (Table 2).

### Dopamine (DA) and its metabolites in the HIP

The Student’s *t*-test revealed an insignificant (*t*_1,20_ = 0.69, *ns*) effect of treatment on DA level in the HIP of adult rats (Table 3). The same analysis showed an insignificant effect (*t*_1,20_ = 0.86, *ns*) on the DOPAC level, and an insignificant effect of treatment (*t*_1,20_ = 1.2, *ns*) on the 3-MT level (Table 3). However, a statistical analysis showed a significant (*t*_1,20_ = 3.61, *p* < 0.01) effect of treatment on HVA levels in the HIP (Table 3).

**Table 3:** The effects of iron (Fe^2+^) administration (30 mg/kg) on the dopamine (DA) metabolism in the hippocampus of 92-day-old rats

Hippocampus					
Treatment	n	DA	DOPAC	3-MT	HVA
Control	12	126 ± 57	24 ± 14	13 ± 5	14 ± 6
Fe^2+^	10	112 ± 25	20 ± 8	17 ± 9	26 ± 8**
*t*-value		*t*_1,20_ = 0.69 *ns*	*t*_1,20_ = 0.86 *ns*	*t*_1,20_ = 1.2 *ns*	*t*_1,20_ = 3.61 *p* < 0.01

The results are expressed as means ± SEM (n = 10–12 animals per group). Data were analyzed with a Student’s *t*-test. Between the postnatal days p12 and p14, male Sprague–Dawley pups were administered orally a single daily dose of vehicle (5% sorbitol of water, control group) or 30 mg/kg of body weight of Fe^2+^ (iron carbonyl). The 92-day-old rats were decapitated, and the tissue of the hippocampus was dissected for biochemical assays. Statistical significance. ** p < 0.01 *vs.* control group

### Noradrenaline (NA) and its metabolite in the HIP

The Student’s *t*-test showed an insignificant (*t*_1,21_ = 1.03, *ns*) effect of treatment on NA level in the HIP of adult rats. Similarly, the effect of treatment on NM level was insignificant (*t*_1,21_ = 0.11, *ns*) (Table 4).

**Table 4:** The effects of iron (Fe^2+^) administration (30 mg/kg) on the noradrenaline (NA) and serotonin (5-HT) metabolism in the hippocampus of 92- day-old rats

Hippocampus					
Treatment	n	NA	NM	5-HT	5-HIAA
Control	12	162 ± 43	19 ± 5	52 ± 18	91 ± 32
Fe^2+^	10	147 ± 27	19 ± 5	40 ± 16	103 ± 36
*t*-value		*t*_1,21_ = 1.03 *ns*	*t*_1,21_ = 0.11 *ns*	*t*_1,21_ = 1.69 *ns*	*t*_1,21_ = 0.82 *ns*

The results are expressed as means ± SEM (n = 10–12 animals per group). Data were analyzed with a Studen’s *t*-test. Between the postnatal days p12 and p14, male Sprague–Dawley pups were administered orally a single daily dose of vehicle (5% sorbitol of water, control group) or 30 mg/kg of body weight of Fe^2+^ (iron carbonyl). The 92-day-old rats were decapitated, and the tissue of the hippocampus was dissected for biochemical assays

### Serotonin (5-HT) and its metabolite in the HIP

Statistical analysis revealed an insignificant (*t*_1,21_ = 1.69, *ns)* effect of treatment on 5-HT amount in the hippocampus of adult rats. The same analysis showed an insignificant effect of treatment (*t*_1,21_ = 0.82, *ns*) on the 5-HIAA level (Table 4).

### Dopamine (DA) and its metabolites in the STR

The Student’s *t*-test showed an insignificant (*t*_1,20_ = 1.49, *ns*) effect of treatment on DA level in the STR of adult rats. The same analysis revealed an insignificant effect (*t*_1,21_ = 0.54, *ns*) on the DOPAC level, and an insignificant effect of treatment (*t*_1,21_ = 1.42, *ns*) on the 3-MT level. A statistical analysis also showed an insignificant (*t*_1,20_ = 1.31, *ns*) effect of treatment on HVA level in the STR (Table 5).

**Table 5:** The effects of iron (Fe^2+^) administration (30 mg/kg) on the dopamine (DA) metabolism in the striatum of 92- day-old rats

Striatum					
Treatment	n	DA	DOPAC	3-MT	HVA
Control	12	6520 ± 1254	3579 ± 883	272 ± 50	1114 ± 192
Fe^2+^	10	5698 ± 1336	3390 ± 775	232 ± 82	1021 ± 126
t-value		*t*_1,20_ = 1.49 *ns*	*t*_1,21_ = 0.54 *ns*	*t*_1,21_ = 1.42 *ns*	*t*_1,20_ = 1.31 *ns*

The results are expressed as means ± SEM (n = 10–12 animals per group). Data were analyzed with a Student’s *t*-test. Between the postnatal days p12 and p14, male Sprague–Dawley pups were administered orally a single daily dose of vehicle (5% sorbitol of water, control group) or 30 mg/kg of body weight of Fe^2+^ (iron carbonyl). The 92-day-old rats were decapitated, and the tissue of the striatum was dissected for biochemical assays

### Noradrenaline (NA) and its metabolite in the STR

The Student’s *t*-test showed a significant (*t*_1,20_ = 2.2, *p* < 0.05) effect of treatment on NA level in the STR of adult rats. At the same time, the effect of treatment on NM level was also significant (*t*_1,20_ = 2.64, *p* < 0.05) (Table 6).

**Table 6:** The effects of iron (Fe^2+^) administration (30 mg/kg) on the noradrenaline (NA) and serotonin (5-HT) metabolism in the striatum of 92- day-old rats

Striatum					
Treatment	n	NA	NM	5-HT	5-HIAA
Control	12	131 ± 47	5 ± 2	76 ± 20	144 ± 38
Fe^2+^	10	87 ± 43*	3 ± 1*	73 ± 19	152 ± 40
t-value		*t*_1,20_ = 2.2 *p* < 0.05	*t*_1,20_ = 2.64 *p* < 0.05	*t*_1,20_ = 0.32 *ns*	*t*_1,20_ = 0.48 *ns*

The results are expressed as means ± SEM (n = 10–12 animals per group). Data were analyzed with a Student’s *t*-test. Between the postnatal days p12 and p14, male Sprague–Dawley pups were administered orally a single daily dose of vehicle (5% sorbitol of water, control group) or 30 mg/kg of body weight of Fe^2+^ (iron carbonyl). The 92-day-old rats were decapitated, and the tissue of the striatum was dissected for biochemical assays. Statistical significance:

*p < 0.05 *vs.* control group

### Serotonin (5-HT) and its metabolite in the STR

Statistical analysis revealed an insignificant (*t*_1,20_ = 0.32, *ns)* effect of treatment on 5-HT amount in the STR. The same analysis showed an insignificant effect of treatment (*t*_1,20_ = 0.48, *ns*) on the 5-HIAA level (Table 6).

### BDNF mRNA expression analysis (real-time PCR)

**Fig. 4:**
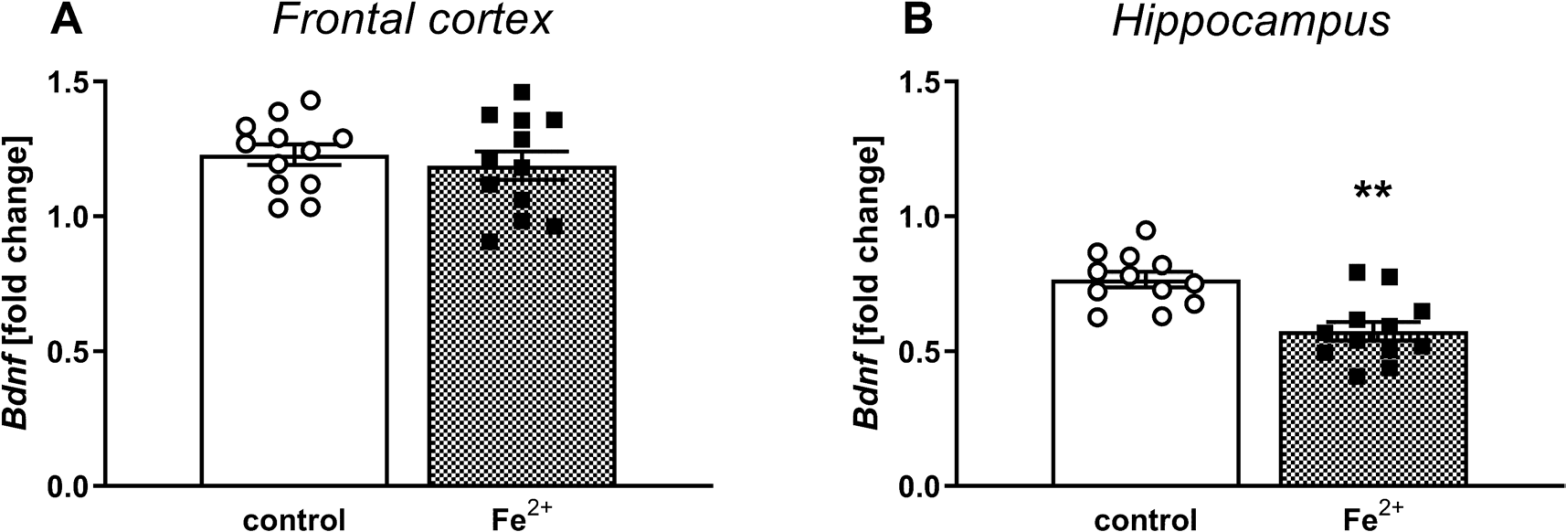
The effects of iron (Fe^2+^) administration on the BDNF mRNA expression in the frontal cortex **(A)** and hippocampus **(B)** in the adult 92 days old Sprague–Dawley rats. Between the postnatal days p12 and p14 male Sprague–Dawley pups were administered orally a single daily dose of 30 mg/kg of Fe^2+^ (iron carbonyl) or vehicle (control group). The results are shown as the mean ± SEM. Each group consisted of 12 rats. The statistically significant differences between the studied groups were calculated using a Student’s *t*-test. ** p < 0.01 *vs.* vehicle-treatment group

The Student’s *t*-test revealed an insignificant (*t*_1,22_ = 0.62, *ns*) effect of treatment on BDNF mRNA expression in the rat’s Fcx of adult rats (Fig. 4A). In contrast, the same analysis showed a significant (*t*_1,22_ = 4.3, *p* < 0.01) effect of treatment on BDNF mRNA expression in the rat’s HIP (Fig. 4B).

## Discussion

In the present study we evaluated the influence of iron administered to Sprague–Dawley male pups in the early postnatal brain development on the performance of adult rats in the behavioral and biochemical tests. It is known that the novel object recognition test is often used to investigate some negative and cognitive symptoms of schizophrenia in animals [25, 26].

The present study indicated that iron administered to rats in the early postnatal brain development induced long-term deficits measured in the behavioral tests in adult rats. In the novel recognition test, it decreased memory retention measured as a shortening time of interest in the new object, and in the social interaction test, iron treatment evoked deficits in the parameters studied, namely, it decreased the time of interactions and the number of episodes. Moreover, it shortened the exploration time in the open field test. The observed behavioral disorders in animals suggest cognitive (memory impairment) and negative (social withdrawal) symptoms of schizophrenia.

Our above data are in line with the previous studies which demonstrated that iron administered to rats in the early postnatal brain development induced a decrease in memory retention evaluated in the novel object recognition test in adult male Wistar rats. Moreover, these authors found that neonatal treatment with iron-induced an increase in oxidative stress via the Fenton reaction which resulted in impaired recognition memory in those rats [16–18].

It was previously demonstrated in biochemical experiments that both DA and NA were involved in functional modulation of the HIP and also other memory-related brain areas [27].

Our present results revealed that iron administered in the neonatal period led to the elevation of the level of HVA (final DA metabolite) in the HIP of adult rats which indicates an increased rate of DA metabolism in this structure, which suggests that the level of DA in the HIP was reduced [21, 28, 29]. Several studies confirm that the activity of DA neurons is important for learning, and disruption of DA transmission is suspected to underlie several psychiatric conditions including schizophrenia, depression, and attention-deficit hyperactivity disorder [30–32]. DA transmission in the lateral prefrontal cortex may be relevant to learning-related cognitive functions [33]. Since the HIP is an important brain structure responsible for memory [34, 35], disturbances in dopaminergic transmission induced by iron in the early stages of development lead to memory impairment, which was confirmed by our observations in the novel object recognition test.

Moreover, our current biochemical study demonstrated that iron administered to rats in the early postnatal brain development induced a decrease in the level of NM (a metabolite of NA) in the Fcx of adult rats, which indicates that the rate of NA metabolism was decreased. Furthermore, a decrease in the level of NA and NM was observed in iron-treated animals’ STR, which clearly **s**howed inhibition of the activity of the entire noradrenergic system. Some data have indicated that NA mediates an arousal-induced memory boost. Thus, NA blockade reduces the implicit arousal-induced memory boost [27]. It has also been shown that chemical lesion of NA neurons leads to impairment of the acquisition of latent learning in the Morris water maze test [36]. Therefore, learning performance is impaired by the reduced NA level [37]. Our present data confirmed that iron administered in the early period of development induced decreases in the activity of the noradrenergic system, which resulted in memory impairment.

The BDNF is the most common neurotrophin in the brain, and it modulates the efficacy of synaptic transmission [38, 39]. There is evidence that BDNF levels are changed in animal models of schizophrenia and other psychiatric disorders [39]. BDNF levels have been shown to be markedly reduced in both plasma and postmortem brains of schizophrenia patients, suggesting that BDNF dysfunction plays a key role in the pathogenesis of this disease. [40].

Moreover, it has been demonstrated that BDNF plays an important role in neuronal development, and synaptogenesis and also as a modulator of monoaminergic and GABAergic neurotransmitter systems [41–44].

Our present data indicated that iron administered in the neonatal period induced a decrease in BDNF mRNA expression only in the HIP, but not in the Fcx in adult rats. It has been demonstrated that the HIP is an important brain structure responsible for memory [34, 35], thus disturbances in dopaminergic transmission induced by iron in the early stages of development may lead to a decrease in BDNF mRNA expression in this structure, and memory deficits, which was confirmed by our present observations in the behavioral and biochemical tests in adult rats.

Some available data indicate that glutathione is widely occurring antioxidant, which plays a key role in the control of the redox state of cells and is involved in controlling gene expression and cell signal transmission. Glutathione deficiency may be associated with disturbances in the dopaminergic, glutamatergic, and GABAergic neurotransmitter systems, which are known to be impaired in schizophrenia [45, 46].

It was previously demonstrated that chronic co-administration of L-butionine-(*S,R*)-sulfoximine (BSO, the major antioxidant and redox regulator in some brain structure) and GBR 12 909 (dopamine reuptake inhibitor) to Osteogenic Disorder Shionogi mutant rats (which like humans cannot synthesize ascorbic acid) in early postnatal brain development, produced a reduction of glutathione level and memory deficits observed in the novel object recognition test in adult rats [47, 48]. The same observations of deficits in behavioral tests, and memory retention tests in adult rats were presented by other authors, after chronic treatment with BSO of Wistar pups [49, 50] or Sprague–Dawley pups in the neonatal period [24]. Moreover, these authors found that neonatal BSO treatment induced an increase in oxidative stress which led to impaired recognition memory in those rats [51–54]. All above results seem to indicate that BSO and GBR 12909 or BSO alone model, which influence cell redox status and dopaminergic transmission, may be a useful as a neurodevelopmental model of schizophrenia.

Recent clinical data have demonstrated a potential relationship between iron overload and schizophrenia symptoms. Brain scans of schizophrenia patients using magnetic resonance imaging have shown elevated iron levels in various brain regions. [55]. Therefore, excess iron in the diet in the early developmental period, when the blood–brain barrier is not yet fully developed, seems to be a good clue to the search for the causes of the development of schizophrenia.

It is worth noting that, animal models, especially those related to mental illnesses, only imitate the symptoms we encounter in humans. And basic research are never 100% applicable to clinical situations. But our preliminary research clearly shows that iron levels in the blood of infants should be monitored before adding iron-fortified formula milk to their diet (corresponding to an iron intake up to 14 mg/day). To avoid iron overdose and, consequently, disturbances in the functioning of the central nervous system in adult life.

In summary, our present findings indicated that iron administered to rats in the neonatal period induced long-term deficits measured in the behavioral tests in adult rats, especially in the social interaction test and novel object recognition test. It is known that these behavioral tests correspond to schizophrenia-like behavior in animals. Moreover, it was earlier demonstrated that iron administered to rats in the neonatal period induced an increase in oxidative stress which resulted in impaired recognition [15, 16]. In our study, iron administered to rats in the neonatal period also induced deficits in behavioral tests and memory retention, changed brain monoamine levels in some studied brain structures, especially in the dopaminergic and noradrenergic systems, and decreased the expression of BDNF mRNA in the HIP in adult rats. All earlier and our present results indicated that iron administered to rats in the neonatal period induced an increase in oxidative stress leading to changes in the brain monoamine levels and decreased BDNF mRNA expression which may play a role in iron-induced memory impairment in adult rats. Further studies are needed to explain the mechanism of action of iron treatment in the neonatal period in the rat model of schizophrenia in adult animals, especially in the prepulse inhibition of the startle response (PPI) test and other models used for studies of the pathomechanism of schizophrenia, which have never been tested in adult rats given iron in the neonatal period of life.

Limitations: The limitation of this study is that only male rats were used for testing and the paucity of literature.

Narrowing the scope of the study to one gender (males) results from a conscious choice made by our team of researchers, which would limit the dispersion of results (especially in the case of behavioral tests). Our decision was due to the fact that in the case of females it is necessary to additionally monitor the hormonal cycle, which is an additional complication. It should be emphasized that these are only preliminary studies and the results obtained should be confirmed in subsequent experiments, which will be performed with both groups of females and males.

## Data Availability

The datasets generated during and/or analyzed during the current study are not publicly available but are available from the corresponding author on reasonable request.

## Abbreviations

BDNF: Brain-derived neurotrophic factor
BSO: L-buthionine-(*S,R*)-sulfoximine
DA: Dopamine
DNA: Deoxyribonucleic acid
DOPAC: 3,4-Dihydroxyphenylacetic acid
Fe^2+^: Iron (carbonyl iron)
Fcx: Frontal cortex
GABA: Gamma-aminobutyric acid
GBR12909: 1-[2-[Bis-4(fluorophenyl)methoxy]ethyl]-4–3-(3-phenylpropyl) Piperazine
HIP: Hippocampus
HVA: Homovanillic acid
5-HIAA: 5-Hydroxyindoleacetic acid
HPLC: High-performance liquid chromatography
5-HT: Serotonin
3-MT: 3-Methoxytyramine
MPAN: Mitochondrial membrane protein associated
NA: Noradrenaline
NBIA: Neurogeneration with brain iron accumulation
NM: Normetanephrine
NMDA: *N*-Methyl-D-aspartate acid
PKAN: Pantothenate kinase-associated neurodegeneration
PPI: Prepulse inhibition of the startle response test
RT-qPCR: Quantitative polymerase chain reaction
STR: Striatum
